# Study of the genetic variability of durum wheat (*Triticum durum* Desf.) in the face of combined stress: water and heat

**DOI:** 10.1093/aobpla/plad085

**Published:** 2023-12-09

**Authors:** Latifa Chaouachi, Miriam Marín-Sanz, Francisco Barro, Chahine Karmous

**Affiliations:** Laboratory of Genetics and Cereal Breeding (LR14 AGR01), National Institute of Agronomy of Tunisia, Carthage University, 1082 Tunis, Tunisia; Department of Plant Breeding, Institute for Sustainable Agriculture-Spanish National Research Council (IAS-CSIC), 14004 Córdoba, Spain; Department of Plant Breeding, Institute for Sustainable Agriculture-Spanish National Research Council (IAS-CSIC), 14004 Córdoba, Spain; Laboratory of Genetics and Cereal Breeding (LR14 AGR01), National Institute of Agronomy of Tunisia, Carthage University, 1082 Tunis, Tunisia

**Keywords:** Abiotic stresses, coping mechanisms, juvenile stage, water and heat combined stress

## Abstract

The devastating effects and extent of abiotic stress on cereal production continue to increase globally, affecting food security in several countries, including Tunisia. Heat waves and the scarcity of rainfall strongly affect durum wheat yields. The present study aims to screen for tolerance to combined water and heat stresses in durum wheat at the juvenile stage. Three combined treatments were tested, namely: T0 (100% field capacity (FC) at 24 °C), T1 (50% FC at 30 °C), and T2 (25% FC at 35 °C). The screening was carried out based on morphological, physiological, and biochemical criteria. The results showed that the combined stress significantly affected all the measured parameters. The relative water content (RWC) decreased by 37.6% under T1 compared to T0. Quantum yield (*F*_v/m_) and photosynthetic efficiency (*F*_v/0_) decreased under severe combined stress (T2) by 37.15% and 37.22%, respectively. Under T2 stress, LT increased by 63.7%. A significant increase in osmoprotective solutes was also observed, including proline, which increased by 154.6% under T2. Correlation analyses of the combination of water and heat stress confirm that the traits RWC, chlorophyll b content, *F*_v/m_, proline content, *F*_v/0_ and leaf temperature can be used as reliable screening criteria for the two stresses combined. The principal component analysis highlighted that Aouija tolerates the two levels of stresses studied, while the genotypes Karim and Hmira are the most sensitive. The results show that the tolerance of durum wheat to combined water and heat stress involves several adaptation mechanisms proportional to the stress intensity.

## Teaser

This study aims to select contrasting drought- and heat-stress-tolerant durum wheat genotypes from a Landraces gene pool using physiological and biochemical traits. The results showed that landraces have a much greater genetic tolerance than the improved variety to mitigate combined stress constraints. Indeed, the landraces Aouija and Hedhba were identified as tolerant to drought and heat stress. Besides, the improved variety Karim and the landrace Hmira are the most sensitive to stress conditions. Our data showed that chlorophyll fluorescence parameters, relative water content, chlorophyll b content, proline content, and leaf temperature can be used as reliable screening criteria for the two stresses combined.

## Introduction

Durum wheat is grown mainly in the Mediterranean basin countries (Koubaa *et al.* 2019), where it occupies an important place in dietary habits, including that of Tunisians (couscous, bourgoul, bsissa, etc.). The Mediterranean region is characterized by: (i) low and erratic rainfall leading to severe droughts; and (ii) rising temperatures during flowering periods (Benkolli and Bouzeghaia 2016). This situation is aggravated by the advent of climate change, which seriously threatens the sustainability of durum wheat cultivation and cereals in general (United Nations Department 2022).

The increase in world population and changes in dietary habits in regions are creating new tensions in agricultural markets (Wang and Luthe 2003; Popović *et al.* 2020; Kavhiza *et al.* 2022; Kiran *et al.* 2022). Cereal production must respond to a rapid change in demand, both quantitatively and qualitatively. In Tunisia, cereal production is characterized by jagged fluctuations (Sourour and Zoubeida 2016). The expansion of unfavourable climatic conditions in cereal-growing areas and the intensification of agricultural practices constitute major abiotic constraints on crops (Bouchemal *et al.* 2017), which reduced yields in durum wheat by 25 quintals per hectare in 2019 to 2 quintals per hectare in 2020 (DGPA 2021).

Drought and heat stresses are the major factors limiting wheat productivity and yields (Mortier 2020). Indeed, the two abiotic stresses cause reductions in the number and weight of grains, inducing significant losses in the yield and quality of cereals (Firmansyah and Argosubekti 2020).

To cope with these constraints and to survive, plants have evolved different adaptation mechanisms by modifying their metabolic systems (Labgaa Nadjat 2018). Currently, besides productivity, which has been the primary objective of most durum wheat improvement (Saidi 2018), breeding programs focus on the genetic improvement of abiotic stress tolerance (drought, salinity, cold, heat stress, etc.) through the identification of morpho-physiological and biochemical parameters (Ghanem 2017). Several physiological, morphological, and biochemical criteria have thus been identified (Benkadja *et al.* 2022). Proline and soluble sugar accumulation, vegetative growth, and photosynthetic efficiency have been considered as tolerance criteria (Boussakouran *et al.* 2019; Jallouli *et al.* 2019; Othmani *et al.* 2019; Gholamin and Khayatnezhad 2020; Pour-Aboughadareh *et al.* 2020; Ayed *et al.* 2021; Tefera *et al.* 2021; Chaouachi *et al*. 2023; Quagliata *et al.* 2023). This research has generated results on the physiological processes related to the tolerance to combined stresses in durum wheat (Benkadja *et al.* 2022). Most studies over the past decade have focused on crop responses to single stress (Chew and Halliday 2011; Siddiqui *et al.* 2015). However, crops often face a combination of stresses in the field (Suzuki and Mittler 2006; Baxter *et al.* 2014; Qaseem *et al.* 2019). In addition, drought and heat stress are often linked and concomitantly cause more losses than when they occur alone (Shah and Paulsen 2003; Dreesen *et al.* 2012). The effect of combined ‘drought/heat’ stress has been studied in several species at different levels: phenological (Mahrookashani *et al.* 2017; Lawas *et al.* 2018), physiological (Pradhan *et al.* 2012; Mahrookashani *et al.* 2017) and metabolic (Rizhsky *et al.* 2004; Templer *et al.* 2017; Lawas *et al.* 2018). In the field, the combined ‘drought/heat’ stress causes a unique plant response that differs from single stresses (Zandalinas *et al.* 2018). The extent of plant damage depends on the severity and duration of the stresses and the stage of growth exposed to them.

Studies have also reported cultivar-specific responses, allowing the identification of genotypes with potential drought and heat stress tolerance (Awasthi *et al.* 2017; Hussain *et al.* 2019; Zhou *et al.* 2020). Nevertheless, few studies have focused on the combined effects of abiotic stresses.

This research study constitutes a further contribution to the efforts aimed at understanding the genetic variability of durum wheat to combined water and heat stresses, making it possible to identify the mechanisms of tolerance deployed and use them in the selection of resilient varieties.

## Material and Methods

### Plant material and growing conditions

The plant material used consisted of five durum wheat (*Triticum turgidum* ssp. *durum*) genotypes, including four Tunisian landrace genotypes (Hmira, Biskri, Hedhba, and Aouija) from the Tunisian National Gene Bank (BNG) and an improved variety (Karim) from the Central Seed Mutual Fund company (COSEM) in Manouba, Tunisia. These genotypes have been previously reported for screening for drought stress conditions (Chaouachi *et al.* 2023).

The seeds of the different genotypes were disinfected with 5% sodium hypochlorite (NaOCl) for 10 minutes, then rinsed three times with distilled water. Seeds were germinated on Wattman filter paper and placed in Petri dishes for 24 hours. The germinated seeds were transplanted into plastic pots (2 L) containing a subtracted mixture of peat and perlite (2:1; v/v) at the rate of three plants per pot. A completely randomized experimental design was adopted, with three replicates for each genotype. The plants were grown under controlled conditions of 21 ± 2 °C (night/day), a photoperiod of 16/8 h and an average humidity of 60%. After 15 days of growth, a test combining water stress (14 days), followed by 2 days of heat stress, was carried out. Three combinations were tested, namely: (i) a control combining the optimal growth conditions of 100% field capacity (FC) at 24 °C (T0); (ii) moderate stress (T1): combining 50% FC at 30 °C; and (iii) severe stress, which combines 25% FC at 35 °C (T2).

### Morpho-physiological traits

Several morphophysiological parameters were evaluated. Thus, leaf area (LA) was measured using ImageJ software (version 1.46r) (Wayne Rasband, NHI, USA). Leaf temperature (LT) was measured by Helect-type infrared thermometry (built-in red laser for precise aiming, Spot Ratio: 12:1, China). Additionally, chlorophyll fluorescence components such as initial fluorescence (*F*_0_), maximum fluorescence (*F*_m_), variable fluorescence (*F*_v_), maximum fluorescence efficiency (F_v/m_), and photosynthetic capacity of leaves (*F*_v/0_) were evaluated using an Opti-Science 30 (Opti-Sciences Inc., USA).

To determine the chlorophyll a and b content, durum wheat leaves were weighed immediately after sampling (~0.5 g) at the rate of one leaf per seedling and then placed in tubes containing acetone (85%). The extraction of the pigments was done based on the Arnon (1949) method. The leaf samples (0.5 g) were ground in 10 mL of 85% acetone. The cell debris was then removed after centrifugation at 3000 rpm for 10 min, and the supernatant was recovered. The amount of chlorophylls a and b was determined by measuring the absorption of the extract at two different wavelengths, 645 and 663 nm.

The relative water content (RWC) (%) was evaluated for the flag leaf according to Turner (1986). Thus, the leaves were cut at the base of the blade, weighed immediately to determine the fresh weight, and then placed in falcon tubes filled with distilled water and stored in the dark. After 24 hours, the samples were weighed again to obtain the turgor weight. These same samples were then dried in an oven at 55 °C for 48 hours before being weighed again to determine the dry weight. The calculation of RWC was made according to the following formula:


$$\begin{array}{l} RWC(\%)=\frac{\left(FW-DW\right)}{\left(TW-DW\right)}\times 100. \end{array}$$

### Biochemical traits

The biochemical parameters that were evaluated were the proline content (PC) and the soluble sugar content (SSC). First, leaf PC was determined using the Bates *et al.* (1973) method. The plant sample (100 mg) was treated with 40% methanol and then heated at 85 °C for 1 h. Then, 1 mL of extracts was added to a mixture of distilled water, acetic acid, and ninhydrin in proportions of 1.2:6:1.4 (v/v/v). The reaction mixture was then incubated in a water bath for 30 minutes at 100 °C. After cooling, the mixture was then extracted by adding 5 mL of toluene and mixed vigorously for 15–20 s. Then, 3 mL of the upper toluene phase was taken. The optical density of the mixture was read at 520 nm. The leaves’ SSC was also measured according to the method of Shields and Burnett (1960). Thus, 100 mg of leaf were ground with 10 mL of ethanol (80%) and incubated in a water bath for 30 min at 70 °C. After cooling, the extract was centrifuged at 6000 rpm for 10 min. Subsequently, 50 μL of the supernatant was added to 5 mL of anthrone (2%) and 2.45 mL of ethanol (80%). Absorbance was measured at 640 nm.

### Data and statistical analysis

For the statistical analysis of the collected data, R (v.3.6.1) was used (Core Development Team R 2020). The effect of water stress treatments on the genotypes studied was determined by a multivariate analysis of variance test (MANOVA). Post hoc analysis was performed using Tukey’s multiple comparison test. All measured variables were used for principal component analysis (PCA), which was performed using the FactoMineR (Lê *et al.* 2008) and Factoextra R libraries (Kassambara and Mundt 2017). In addition, agglomerative hierarchical clustering, using the unweighted pair group method with the arithmetic mean method (UPGMA), was performed to rank the genotypes according to their tolerance.

## Results

### Effect of combined stress on morpho-physiological and biochemical traits

In this study, nine morpho-physiological and biochemical traits were used to screen the water stress tolerance of durum wheat genotypes. All these traits were under the significant effect of the genotype × combined stress treatments (*G* × *T*), except LA (Table 1).

**Table 1.** Averages of the parameters measured: LA (cm^2^), proline content (µg/g of fresh material (MF) (PC), SSC (µg/g of FM), quantum yield (*F*_v/m_), photosynthetic efficiency (*F*_v/0_), the LT (°C), relative water content (%) (RWC), the chlorophyll a content (μg/mL) (Chla), and the chlorophyll b content (μg/mL) (Chlb), for the five durum wheat genotypes conducted under combined stress treatments (water and heat): T0: 100% FC_24 °C, T1: 50% FC_30 °C, and T2: 25% FC_35 °C. ANOVA (top) and MANOVA (bottom) results are shown. Tukey’s test was performed to compare the average of the treatments. *P < 0.05; **P < 0.01; ***P < 0.001; NS, non significant.

Genotypes (G)	Treatments (T)	LT	*F*_v/m_	*F*_V/0_	Chla	Chlb	RWC	LA	SSC	PC
Aouija	T0	23.8 a	0.74 a	4.70 a	13.09 a	8.49 a	94.04 a	54.3 a	1.22 a	0.158 a
	T1	27.5 b	0.72 a	3.94 b	9.87 ab	7.83 a	72.04 b	51.0 a	2.77 b	0.332 b
	T2	35.5 c	0.61 b	3.13 c	8.53 b	5.26 b	40.56 c	26.0 b	3.69 c	0.474 c
Hedhba	T0	22.9 a	0.72 a	3.11 a	14.70 a	6.89 a	95.44 a	76.3 a	1.49 a	0.097 a
	T1	28.2 b	0.64 a	2.91 ab	10.85 b	6.83 a	61.08 b	68.4 a	3.28 b	0.173 a
	T2	35.3 c	0.47 b	2.80 b	9.42 b	3.05 b	33.75 c	60.2 b	3.54 b	0.195 b
Hmira	T0	23.5 a	0.80 a	3.12 a	19.74 a	9.81 a	99.12 a	54.7 a	1.49 a	0.087 a
	T1	29.0 b	0.56 b	1.40 b	15.92 b	6.58 b	48.14 b	42.4 ab	2.32 b	0.153 b
	T2	38.8 c	0.42 c	1.35 b	10.30 a	2.82 c	25.23 c	35.4 b	2.59 b	0.206 b
Karim	T0	23.3 a	0.79 a	3.07 a	15.11 a	10.80 a	91.35 a	49.0 a	1.74 a	0.083 a
	T1	31.6 b	0.46 b	1.97 b	6.90 b	6.82 b	54.44 b	41.5 ab	2.38 ab	0.169 b
	T2	38.1 c	0.38 c	2.00 b	4.72 b	3.75 c	20.84 c	28.8 b	2.63 b	0.180 b
Biskri	T0	23.4 a	0.76 a	2.64 a	12.17 a	8.99 a	95.85 a	55.7 a	1.77 a	0.097 a
	T1	28.3 b	0.63 b	1.73 b	9.03 ab	4.29 b	60.81 b	39.7 ab	2.61 b	0.173 b
	T2	37.2 c	0.53 c	1.63 b	8.53 b	3.07 b	33.73 c	23.3 b	3.00 b	0.195 b
Genotypes (*G*)		***	***	***	***	***	***	***	***	***
Treatments (*T*)		***	***	***	***	***	***	***	***	***
*G* × *T*		**	***	***	**	***	***	NS	**	***

**Figure 1.**
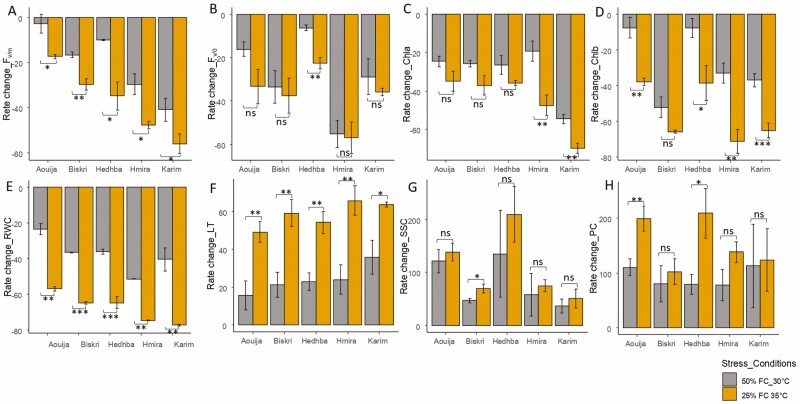
Rate change of eight measured variables of five durum wheat genotypes subjected to two levels of combined stress (heat and water stresses), namely: T1: 50% FC at 30 °C and T2: 25% FC at 35 °C, evaluated in pots at the juvenile stage (Z14). The measured traits are LA, PC, SSC, quantum yield (*F*_v/m_), photosynthetic efficiency (*F*_v/0_), LT, RWC, chlorophyll a content (Chla), and chlorophyll b content (Chlb). *, *P* < 0.05; **, *P* < 0.01; ***, *P* < 0.001; NS, non-significant.

The results revealed that the photosynthetic efficiency (*F*_v/0_) decreased under combined stress compared to the control. Under moderate stress (T1), *F*_v/0_ decreased by 28.02% compared to a decrease of 37.22% under severe stress (T2) compared to the control (T0) (Fig. 1B).

The quantum yield (*F*_v/m_) was significantly reduced by the combined stress for all the studied genotypes. The highest rate of decrease was observed under T2 for the genotype Karim (52.02%) compared to the control, while Aouija was the least affected (Fig. 1A).

Chlorophyll a and b content tends to decrease under combined stress. Thus, under moderate stress (T1), the chlorophyll content decreased by 30.1%, compared to a decrease of 44.9% under severe stress (T2) (Fig. 1C). Moreover, only the genotypes Karim and Hmira showed a significant decrease in Chla under both stress levels (T1 and T2) (Fig. 1D). Interestingly, the Chla/Chlb ratio decreased for all the tested genotypes except for the genotype Karim. Indeed, the greatest decrease was observed in the genotypes Biskri (105.3%) and Hmira (81.5%) under T2 compared to the control. However, in the genotype Karim, the Cha/Chlb ratio was about 10% under T2 compared to under T0. It turned out that the chlorophyll pigments a and b were not affected to the same extent in the different genotypes. Thus, for the genotype Karim, the Chlb content was much more affected than the Chla under T2 treatment (Table 1).

A general trend of decreasing RWC was noted in the durum wheat genotypes studied (Fig. 1E). Indeed, the maximum reduction in RWC was recorded under severe stress (T2) (67.6%), against a decrease of 37.58% under moderate stress (T1), compared to T0. Importantly, the genotype Karim possesses the most remarkable reduction in RWC (77.2%) compared to other genotypes under severe combined stress (T2) (Fig. 1E).

Genotypic variability was observed for LT, with a general trend of increase under combined stress compared to control (T0) (Fig. 1F). Under moderate stress (T1), LT increased by 16.97% compared to an increase of 26.03% under severe stress (T2) compared to control (T0) (Fig. 1F). The highest mean LT was observed in the genotypes Hmira and Karim at 38.8 °C and 38.1 °C, respectively (Table 1).

Abiotic stress caused a reduction in LA of 16.20% and 41.4% under moderate (T1) and severe (T2) stress, respectively. The greatest decrease was observed under severe stress (T2) for Biskri (57.3%) and Aouija (32.9%) (Table 1).

The applied stress resulted in an accumulation of soluble sugars (SSC) proportional to the severity of the combined stress. The greatest increase in SSC was noted for Aouija under T2 with a 179% increase over the control (T0) (Fig. 1G). The maximum increase was recorded under severe stress (T2), with an increase of 102.5%, followed by an increase of 77.2% under moderate stress (T1).

The variation in PC was significantly (*P* < 0.001) affected by the different treatments applied (Table 1). Thus, PC tends to increase under the effect of combined stress. The maximum increase was recorded under severe stress (T2) with 154.6% and under moderate stress (T1) with 92.2%, compared to the control (T0). The results showed that the genotype Hedhba has the highest PC (209.1%) under the two stress combinations, followed by Aouija with 199.6% (Fig. 1H).

To study the degree of the relationships between the measured parameters, a Pearson correlation analysis (*α* = 5%) was carried out for the combined stress (water and heat).

**Figure 2.**
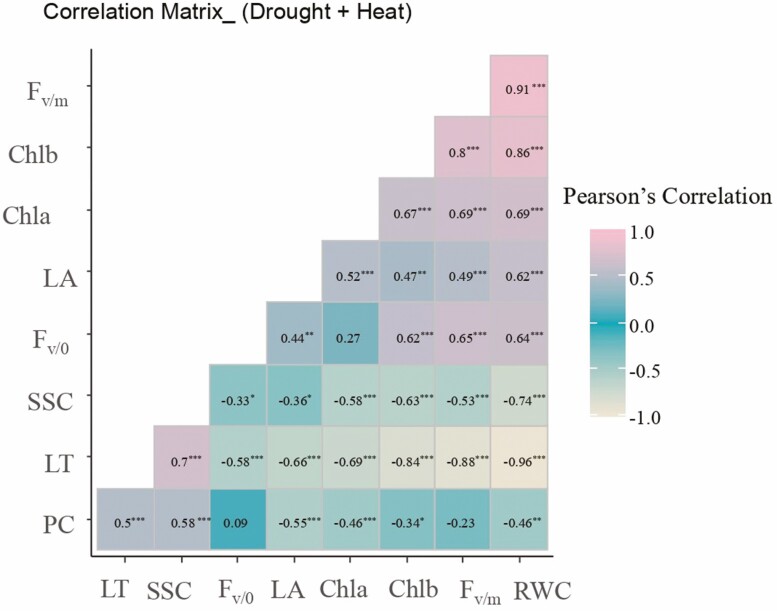
Graphic representation of the Pearson correlations (*α* = 5%) of the measured parameters of five durum wheat genotypes in the face of combined stress (heat and water). Such as LA, PC, SSC, quantum yield (*F*_v/m_), photosynthetic efficiency (*F*_v/0_), LT, RWC, chlorophyll a content (Chla), and chlorophyll b content (Chlb). *, *P* < 0.05; **, *P* < 0.01; ***, *P* < 0.001; NS, non-significant.

Correlation analysis (Fig. 2) highlighted positive correlations recorded between RWC and the chlorophyll fluorescence traits *F*_v/m_ and *F*_v/0_ (*r* = 0.91^***^, *r* = 0.64^***^, respectively). Also, a positive correlation was observed between RWC and Chla (*r* = 0.69^***^) and Chlb (*r* = 0.86^***^), confirming the importance of RWC in the components of photosynthesis mechanisms under harsh environmental conditions. Interestingly, RWC was shown to be negatively correlated with the osmo-regulators, which tend to increase under stress conditions, SSC and PC (*r* = −0.74^***^ and *r* = −0.46). Moreover, *F*_v/m_ was positively correlated with *F*_v/0_, Chla, and Chlb (*r* = 0.65^***^, *r* = 0.69^***^, *r* = 0.8^***^). Positive associations were recorded between Chlb and Chla (*r* = 0.67***) and *F*_v/0_ (*r* = 0.62^***^). As expected, a positive correlation was recorded between SSC and PC (*r* = 0.58^***^). Regarding the LT, this turned out to be positively correlated with PC and SSC (*r* = 0.5***, *r* = 0.7^***^, respectively). On the other hand, the negative effect of the high temperature was translated by a negative correlation between LT and RWC, *F*_v/m_, *F*_v/0_, Chla, Chlb, and LA with (*r* = −0.96^***^, *r* = −0.88^***^, *r* = −0.84^***^, *r* = −0.58, *r* = −0.69^***^, r = −0.84***, and *r* = −0.66^***^, respectively). LA was negatively correlated with PC (*r* = −0.55^***^). Negative correlations were recorded between *F*_v/m_ and LT (*r* = −0.88^***^) (Fig. 2). As well, Chlb was negatively correlated with SSC (*r* = −0.63^***^) (Fig. 2).

### Grouping and genotypic hierarchical classification of durum wheat under combined stress

**Figure 3.**
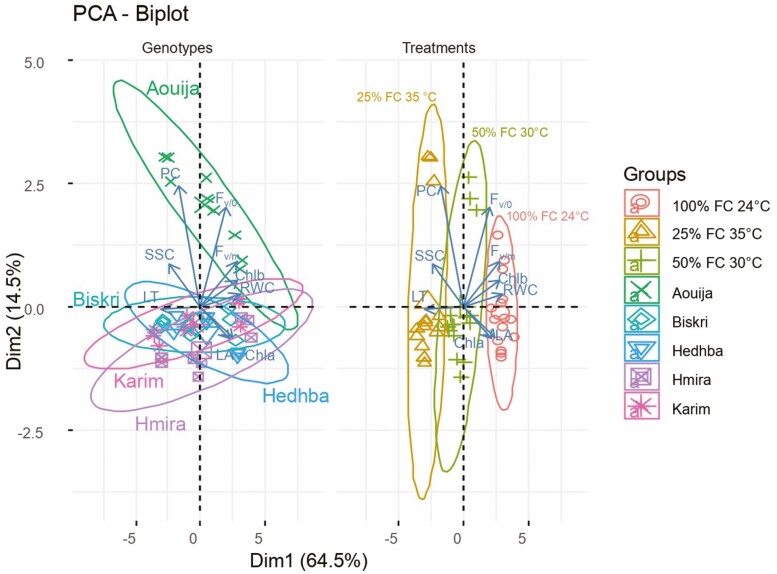
PCA of all the variables measured for the five genotypes and three studied treatments (T0: 100% field capacity (FC) at 24 °C, T1: 50% FC at 30 °C, and T2: 25% FC at 35 °C). Projection of durum wheat genotypes and morpho-physiological and biochemical parameters under three combined water and heat treatments. Ellipses with 95% confidence were plotted to allow the grouping of genotypes and treatments.

PCA explained 79% of the cumulative variance of parameters measured for durum wheat under combined stress (Fig. 3).

The PCA biplot allowed the identification of three groups of durum wheat genotypes (Fig. 3). Indeed, the PCA showed that the 95% confidence ellipses of the genotypes Karim, Biskri, and Hmira are narrow, and they are in the same direction (Fig. 3). The second group is formed by the genotype Aouija. Indeed, the ellipse of this group is in the opposite direction to that of the first group. This group is characterized by PC, accompanied by quite significant SSC and *F*_v/0_. The third group is formed by the genotype Hedhba. The second and third groups are narrowing in the same direction. Hence, they probably have the same features.

The traits that contributed the most to PCA were RWC, Chlb, *F*_v/m_, PC *F*_v/0_ and LT. However, Chla, SSC, and LA were the traits that contributed the least to the level of PCA pattern variation (Fig. 3).

**Figure 4.**
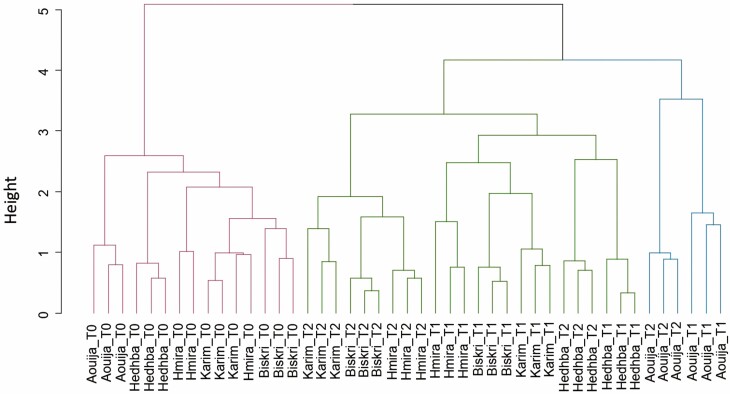
Dendrogram of the mean of all measured parameters and genotypes tested under three combined water and heat stress treatments (T0: 100% FC at 24 °C, T1: 50% FC at 30 °C, and T2: 25% FC at 35 °C).

The hierarchical classification made it possible to classify four groups of genotypes according to the degree of resemblance of measured quantitative data and according to the levels of stress applied (Fig. 4).

The first group contains all the genotypes tested under control conditions (T0). The second group encompasses the classification of the genotypes Karim, Biskri, and Hmira under severe combined stress (T2). In the third group, we find that the genotype Hedhba under T2 was classified with the genotypes Karim, Biskri, and Hmira under severe combined stress (T2). The fourth group is formed by the genotype Aouija under the two stress levels (T1) and (T2) (Fig. 4).

On this basis, the genotypes Karim, Hmira, and Biskri are relatively sensitive to severe combined stress (T2), while Aouija and Hedhba are potentially tolerant of combined stress.

## Discussion

Plants respond to stress through morphological, physiological, and biochemical adaptations (Sagar *et al.* 2022). The great variability of responses was noted for all the parameters measured. Indeed, the effect of stress depends on its degree, duration, the stage of plant development, the genotype, and its interaction with the environment (Maazouz Ahlem 2021).

Our results showed that the lack of water accompanied by high temperatures induced a significant decrease in RWC, LA, and chlorophyll rate, which is in agreement with the findings of Bakha *et al.* (2019). This situation fosters more accelerated senescence and leaf abscission (Kosová *et al.* 2011). Indeed, a reduction in the hydration of durum wheat induces a decrease in biomass (Benziouche Achouak 2021). Plant water status is highly dependent on thermal stability. An excessive increase in heat causes dehydration of plant tissues, which limits the growth and development of plants (Benhamed and Benamara 2020). The water retention capacity of the foliage is suggested to assess the resistance of plants to heat stress and seems to be a good indicator of genotypic resistance to abiotic stresses (Saidi 2018).

Leaf water status, measured by leaf RWC, is widely used for abiotic stress assessments (Zhou *et al.* 2017; Tani *et al.* 2019). Leaf RWC decreased rapidly under combined drought and heat stress in *Camellia oleifera* (Wang *et al.* 2015), perennial ryegrass (*Lolium perenne*) (Jiang and Huang 2001) and wheat (Jiang and Huang 2001; Qaseem *et al.* 2019). Thus, RWC can be used as a useful indicator to select wheat genotypes with potential drought tolerance. The maximum reduction in RWC was obtained under severe combined stress (T2), essentially in the genotype Karim. Several studies have shown that wheat genotypes with reduced leaf water loss are considered more drought-tolerant (Lamaoui *et al.* 2018; Ayed *et al.* 2021).

In addition, drought and heat stress can induce leaf senescence, leading to chlorophyll degradation and photosynthesis disruption (Wang *et al.* 2018), which hamper crop yield. Chlorophyll is the main pigment for photosynthesis and is most sensitive to high temperatures (Wang *et al.* 2015) and water stress (Oneto *et al.* 2016). High temperatures destroy chlorophyll and damage plants by reducing light quantum acceptance (Zafar *et al.* 2017). Similarly, chlorophyll concentrations decreased more in plants exposed to combined drought and heat stress than individual stresses in wheat (Prasad *et al.* 2011; Farooq *et al.* 2017; Qaseem *et al.* 2019). A decrease in chlorophyll a and b pigments under combined drought and heat stresses was reported in a stress-sensitive wheat cultivar (Abdelhakim *et al.* 2021). Nonetheless, the calculation of the Cha/Chlb ratio under severe drought and heat stress revealed that the reduction of the two pigments was not in an equal way in all the genotypes. Indeed, Biskri and Hmira had the highest decrease in Chla/Chlb under T2 treatment. Thus, for example, Hmira had the least reduction of Chla but the highest decrease of Chlb. This means that the effect of combined stress on the biosynthesis of chlorophyll pigments depends on the genotypic component. These results are in accordance with those found by Sattar *et al.* (2020) and Abdelhakim *et al.* (2021).

On the other hand, combined drought and heat stress affect chlorophyll fluorescence in various crops, reducing or even stopping PSII activity due to a reduced concentration of photosynthetic pigments (Bhardwaj *et al.* 2021). For example, a combined drought/heat stress (35/20 °C; 40–45% FC) applied for 15 days significantly reduced chlorophyll fluorescence values in 12 wheat varieties (Balla *et al.* 2006).

In addition, chlorophyll fluorescence analysis revealed that applied stress results in a significant decrease in *F*_v/m_ for all studied genotypes. This decrease can be explained by a reduction in the transfer of electrons to the reaction centre of the PSII due to variations in energy absorption, electron trapping, transport and dissipation per section, leading to a reduction in photosynthetic efficiency of PSII (Stirbet *et al.* 2018; Khatri and Rathore 2019). According to Aberkane *et al.* (2021), water stress followed by heat stress progressively decreases electron transport from PSII and increases non-photochemical quenching in wheat leaves that support the role of alternative sink electrons (from either PSII or PSI) and the flow of cyclic electrons for the photoprotection of PSII and PSI, which also generate the ATP needed to cope with combined stress conditions. In this sense, the chlorophyll fluorescence of the genotype Aouija was the least affected.

In addition, *F*_v/m_ and *F*_v/0_ are considered important parameters to assess the integrity of internal mechanisms in the leaf during photosynthetic activities and are considered an accurate method for screening plant tolerance to water stress (Zhu *et al.* 2021). Our results showed that these parameters are strongly affected by the applied stresses (Table 1).

A reduction in *F*_v/m_ by water deficit and high temperature suggests a possible inhibition of PSII photochemistry, which may be due to an inadequate relocation of the energy of the light-harvesting chlorophyll complex towards the reaction centre (Ahmed *et al.* 2013). A minimal decrease in *F*_v/m_ was observed for Aouija indicating that higher defensive capacity for PSII is a key resistance mechanism for durum wheat landraces, which is in agreement with previous studies for durum wheat accessions under water and heat stress conditions (Marček *et al.* 2019; Doneva *et al.* 2021; Chaouachi *et al.* 2023; Raza *et al.* 2023). For the parameters reported in this work, Aouija is more tolerant to abiotic stress and could be an opportunity for breeding programs. It is important to remember that these morpho-physiological parameters were defined by low rates of decrease in the genotypes Aouija and Hedhba, which suggests that the latter two are tolerant to combined stress despite the accumulation of stress effects.

To survive combined drought/heat stress, plants change their metabolism to promote the synthesis of osmolytes and secondary metabolites that promote stress tolerance (Alhaithloul *et al.* 2019). Plants affected by drought and heat stress accumulate compatible solutes, such as proline, glycine, betaine and soluble sugars, which play a role in osmotic adjustment to maintain water status and protect leaf cells. Osmotic adjustment allows the plant to maintain turgor pressure at low water potential, which is important for maintaining metabolic functions (Izanloo *et al.* 2008; Bhutto *et al.* 2023). The results showed that the genotypes Hedhba and Aouija showed the highest proline accumulation. A high capacity for proline accumulation under water and/or heat stress is generally associated with high stress tolerance (Priya *et al.* 2019; Chaouachi *et al.* 2023; Quagliata *et al.* 2023). Increasing the production of these osmoprotectants decreases ROS production and reduces leaf senescence (Hanif *et al.* 2021). Proline is part of many stress signalling pathways involved in stress adaptation (Qaseem *et al.* 2019). A connection between leaf proline accumulation and yield stability under water and heat stresses has been reported in barley (Oukarroum *et al.* 2012) and wheat (Qaseem *et al.* 2019; Sattar *et al.* 2020).

Under conditions of water and heat stress, plants accumulate other osmotically active substances like sugars (Basu *et al.* 2016; Sami *et al.* 2016; Xie *et al.* 2020; Chunyan *et al.* 2022; Hussein *et al.* 2022). Our data showed a substantial increase in this solute correlated with the severity of applied stress, as previously reported (Adrees *et al.* 2020; Sedaghat *et al.* 2020). The increase in SSC was higher under the treatments at (T2) than under (T1) and this trend was particularly remarkable for Aouija than for the other genotypes. Sensitive plants showed less increase in SSC than tolerant plants (Abid *et al.* 2018).

Our results show that the five durum wheat genotypes use the same strategies to tolerate combined stress (Chaib *et al.* 2015), and emphasize the accumulation of single stresses that induced remarkable damage illustrated by the low growth and the sharp reduction in photosynthetic activity which will also influence production and yield later (Zitouni and Abdi 2014).

## Conclusion

The study of the response to combined water and heat stress in durum wheat confirmed genotypic variability in tolerance to stress applied at the juvenile stage.

Water and heat stresses cause profound morpho-physiological and biochemical changes in the various vegetative organs involved in this study. Indeed, the desiccation of the substrate induces a strong reduction in the RWC of the plant observed in Karim and Hmira followed by Biskri. However, a significant increase in PC was recorded mainly in the genotype Hedhba followed by Aouija under the effects of combined stress.

The results showed that the genotypes Karim and Hmira exhibit an increased sensitivity to combined stress. However, it is the genotype Aouija that showed tolerance stability under the different stress levels tested, which makes this genotype an interesting genetic source for breeding programmes. Indeed, pre-breeding efforts are needed to provide sources of valuable traits that breeders need to develop the idiotype genetic material that will adapt to the effects of climate change.

The results obtained open the way to several other perspectives such as the confirmation of the results obtained at the maturation stage via an evaluation of the impact of water and thermal stress on the flowering and fertilization phase. On the other hand, it seems interesting to complete the work with molecular biology studies to identify the genes involved in the mechanisms of tolerance.

## Supplementary Material

plad085_suppl_Supplementary_Data

## Data Availability

Data are available as Supporting Information (Excel file).
